# Higher omega-3 index is associated with increased insulin sensitivity and more favourable metabolic profile in middle-aged overweight men

**DOI:** 10.1038/srep06697

**Published:** 2014-10-21

**Authors:** Benjamin B. Albert, José G. B. Derraik, Christine M. Brennan, Janene B. Biggs, Greg C. Smith, Manohar L. Garg, David Cameron-Smith, Paul L. Hofman, Wayne S. Cutfield

**Affiliations:** 1 Liggins Institute, University of Auckland, Auckland, New Zealand; 2 Department of Pharmacology, University of New South Wales, Sydney, New South Wales, Australia; 3 Nutraceuticals Research Group, University of Newcastle, Callaghan, New South Wales, Australia; 4 Gravida: National Centre for Growth and Development, Auckland, New Zealand

## Abstract

We assessed whether omega-3 index (red blood cell concentrations of eicosapentaenoic acid (EPA) and docosahexaenoic acid (DHA)) was associated with insulin sensitivity and other metabolic outcomes in 47 overweight men aged 46.5 ± 5.1 years. Participants were assessed twice, 16 weeks apart. Insulin sensitivity was assessed by the Matsuda method from an oral glucose tolerance test. Linear associations were examined; stratified analyses were carried out with participants separated according to the omega-3 index: lower tertiles (LOI; n = 31) and highest tertile (HOI; n = 16). Increasing omega-3 index was correlated with higher insulin sensitivity (r = 0.23; p = 0.025), higher disposition index (r = 0.20; p = 0.054), and lower CRP concentrations (r = −0.39; p < 0.0001). Insulin sensitivity was 43% higher in HOI than in LOI men (Matsuda index 6.83 vs 4.78; p = 0.009). Similarly, HOI men had disposition index that was 70% higher (p = 0.013) and fasting insulin concentrations 25% lower (p = 0.038). HOI men displayed lower nocturnal systolic blood pressure (−6.0 mmHg; p = 0.025) and greater systolic blood pressure dip (14.7 vs 10.8%; p = 0.039). Men in the HOI group also had lower concentrations of CRP (41% lower; p = 0.033) and free fatty acids (21% lower, p = 0.024). In conclusion, higher omega-3 index is associated with increased insulin sensitivity and a more favourable metabolic profile in middle-aged overweight men.

Insulin resistance, defined as a pathological reduction in insulin sensitivity, has an important role in the pathogenesis of essential hypertension, dyslipidaemia, and type 2 diabetes mellitus^1^. These conditions are components of the metabolic syndrome, and are major risk factors for cardiovascular and cerebrovascular disease^2^, chronic renal failure^3^, and retinopathy^4^. In addition, insulin resistance may be a risk factor for malignancy^5^. As the rates of overweight and obesity continue to rise, insulin resistance is becoming one of society's most pressing health problems.

Many factors influence insulin sensitivity, including age, adiposity, perinatal factors, and genotype. Lifestyle factors such as diet and physical activity also affect insulin sensitivity, and are particularly important because they are modifiable. Although weight loss^6^ and increasing physical activity^7^ improve insulin sensitivity, these goals are difficult to achieve for a large proportion of the population. In contrast, relatively small dietary modifications (such as supplementation with nutraceuticals or increased consumption of fish) are much easier to attain. Thus, if such dietary modifications can improve insulin sensitivity in at-risk groups, it may be possible to lower the incidence of type 2 diabetes, the metabolic syndrome, and cardiovascular disease in the general population.

There is increasing evidence suggesting that dietary omega-3 polyunsaturated fatty acids (n-3 PUFA), particularly the long-chain fatty acids eicosapentaenoic acid (EPA) and docosahexaenoic acid (DHA) found in marine oils, may improve insulin sensitivity or reduce the incidence of type 2 diabetes. Epidemiological studies have linked higher dietary^8^ or plasma^9^,^10^ n-3 PUFA concentrations with lower risk of diabetes. Rodent studies have also shown that insulin resistance can be reversed by supplementation with fish oil^11^,^12^,^13^. However, human intervention trials have yielded inconclusive results. In a systematic review that included 11 randomized controlled trials and 618 participants, n-3 PUFA supplementation did not influence insulin sensitivity^14^. However, the individual trials were highly heterogeneous, including participants with and without type 2 diabetes, utilising a wide range of n-3 PUFA doses, as well as adopting a range of treatment and control oils. In association with weight loss^15^ or caloric restriction^16^,^17^ supplementation with fish or fish oil increased insulin sensitivity. A study showed that switching to a Mediterranean diet (which includes a lower dietary n-6:n-3 ratio) also increased insulin sensitivity^18^, but it was not possible to isolate the specific effect of n-3 PUFA due to the complexity of dietary interventions. In a simple dietary intervention trial of 12 healthy older adults, changing from non-oily fish to oily fish improved insulin sensitivity^19^. However, in the multicentre KANWU study, addition of fish oil to a high saturated fat or high monounsaturated fat diet did not influence insulin sensitivity^20^.

The relationship between insulin sensitivity and n-3 PUFA (EPA/DHA) concentrations in red blood cells (omega-3 index^21^) has not been previously examined in adulthood. If higher blood n-3 PUFA concentrations are associated with improved insulin sensitivity, this would provide a mandate for larger and better controlled interventional studies. Thus, we examined the association of omega-3 index with insulin sensitivity and other metabolic indices, in a cohort of overweight middle-aged men enrolled into a randomised clinical trial.

## Methods

### Ethics

Ethical approval was granted by the Central Regional Ethics Committee, New Zealand Ministry of Health (CEN/11/07/038). Written and verbal informed consent was obtained from all participants. This study was performed in accordance with all appropriate institutional and international guidelines and regulations for medical research, in line with the principles of the Declaration of Helsinki.

### Participants

Volunteers were recruited in 2012 using advertisements in local newspapers that circulate freely in the central Auckland metropolitan area. Overweight (body mass index (BMI) 25–30 kg/m^2^), middle-aged (35–55 years) men were eligible to participate. The study cohort represents a higher risk group, likely to have early insulin resistance without clinical disease, enabling easier detection of important factors. Note that only males were recruited, so that the effects of the menstrual cycle and/or oral contraceptives on insulin sensitivity (the primary outcome) could be avoided. Exclusion criteria were: diabetes mellitus, hypertension (systolic blood pressure > 145 mmHg or diastolic > 95 mmHg), known dyslipidaemia, the use of tobacco, or prescription medications likely to affect blood pressure, lipid profile or insulin sensitivity. Participants taking fish oil or other omega-3 supplements were asked to stop supplementation 4 weeks prior to the first assessment.

### Study design

All participants were assessed on two occasions, 16 weeks apart, corresponding to the baseline samples of a crossover clinical trial. Importantly, there were no differences in omega-3 index between the two assessments. Clinical assessments were carried out between 07:00 and 09:00 at the Maurice & Agnes Paykel Clinical Research Unit (Liggins Institute, University of Auckland), after an overnight fast and no strenuous activity over the previous 24 hours.

The primary outcome was insulin sensitivity measured with a 75 g oral glucose tolerance test (OGTT) using the Matsuda method. Blood samples were collected at 0, 30, 60, 90, and 120 minutes^22^ for glucose and insulin measurements. The Matsuda index has a strong correlation with the hyperinsulinemic euglycaemic clamp (r = 0.77)^23^, and excellent reproducibility during multiple measures^24^. The oral disposition index (a measure of β-cell function corrected for insulin sensitivity) was also calculated^25^. During the OGTT, blood was collected into collection tubes containing DPP-IV inhibitor at all time points measured. The active GLP-1 concentrations during the OGTT were used to calculate the area under the curve (AUC).

Fasting blood samples were also used to measure n-3 and n-6 PUFA concentrations in red blood cells. These concentrations reflect assimilation of dietary PUFA over a longer period of time than those in plasma^26^,^27^, so that they are less prone to short-term fluctuation. Further, as this measure reflects lipid concentrations in the cell membrane, it may be more relevant than plasma concentration to physiological processes such as eicosanoid synthesis.

In addition, fasting samples were used to assess other measures of metabolic disease risk, including uric acid, free fatty acid, and highly-sensitive C-reactive protein (CRP) concentrations, as well as lipid profile (triglyceride, total cholesterol, high-density lipoprotein cholesterol (HDL-C), and low-density lipoprotein cholesterol (LDL-C) concentrations). Auxological assessment included height measurement using a Harpenden stadiometer. Weight and body composition were assessed using whole-body dual-energy X-ray absorptiometry (DXA, Lunar Prodigy 2000, General Electric, Madison, USA).

24-hour ambulatory blood pressure monitoring was carried out prior to each clinical assessment. Participants were fitted with a Spacelabs 90207 or 90217 monitor (Spacelabs Medical Inc., Redmond, USA), with each subject being assigned the same device model for all assessments. Measurements were performed every 20 minutes between 07:00 and 22:00, and every 30 minutes from 22:00 to 07:00. Only profiles with >14 daytime and >7 night time recordings over a 24-hour period were analysed^28^.

Carotid artery intima-media thickness was also measured to assess possible treatment effects, as it is a validated and reproducible measure that is predictive of cardiovascular and cerebrovascular risks^29^. Carotid intima-media thickness was measured using an M-Turbo ultrasound system (Sonosite, Bothel, USA) by a trained investigator [BBA], with longitudinal images attained using a standard protocol^30^. The right common carotid artery was scanned from both posterolateral and anterolateral views. Digitally stored images were analysed using computer software automated callipers to measure the far wall (SonoCalctm v.4.1, Sonosite). The maximal thickness measurement from both views (approximately 10 mm proximal to the carotid bulb) was used for comparative analysis. To assess reproducibility, triplicate measures were taken of seven healthy volunteers over a 7-day interval, and resulted in an intra-observer CV of 3.7% (unpublished data).

Lifestyle factors were recorded with an itemised food diary and physical activity recall. Three-day dietary records were collected prior to clinical assessments. Each dietary report encompassed an itemized nutritional intake recorded during two week days (Monday to Friday) and one weekend day. Nutritional intake was recorded using standard household measures, as well as the information from food labels where appropriate. Participants were instructed by a trained investigator [BBA], who also reviewed all food records with each participant to address unclear descriptions, errors, omissions, or doubtful entries. Records were subsequently entered into Foodworks software (v6.0, Xyris Software, Brisbane, Australia) by the trained investigator [BBA]. Physical activity levels were assessed using the International Physical Activity Questionnaire (IPAQ)^31^, covering four domains of physical activity: work-related, transportation, housework/gardening, and leisure time.

Geo-coded deprivation scores were derived from current address using the New Zealand Index of Deprivation 2006 (NZDep2006)^32^. This index is based on household census data reflecting nine aspects of material and social deprivation to divide New Zealand into tenths (scored 1–10) by residential address. Scores of 1 represent the least deprived areas and 10 the most deprived. Scores are derived from units covering a small area, each reflecting approximately 90 people. Ethnicity was recorded by self-report using a prioritised system, such that if multiple ethnicities were selected, the patient was assigned to a single category, following a hierarchical system of classification.

### Assays

Insulin concentrations were measured using an Abbott AxSYM system (Abbott Laboratories, Abbott Park, USA) by microparticle enzyme immunoassay with an inter-assay coefficient of variation (CV) of 5.4%. Glucose, triglyceride, total cholesterol, HDL-C, LDL-C, free fatty acid, uric acid, and highly-sensitive CRP concentrations were also measured on a Hitachi 902 autoanalyser (Hitachi High Technologies Corporation) by enzymatic colorimetric assay (Roche) with all CVs lower than 3.2%. Active GLP-1 levels were quantified using ELISA kits (Millipore); this assay had a CV of 7.2%.

### Erythrocyte fatty acid analysis

Fatty acid profile was analysed via direct transesterification of the washed erythrocyte (RBC) fraction of blood, followed by gas chromatography^33^. Methanol:toluene 2 ml (4:1 v/v) (containing C19:0 (20 μg/ml) as internal standard) was added to the sample. Acetyl chloride (200 μl) was added while vortexing and then heated (1 hour, 100°C). The tubes were then cooled in water (5 minutes), had K_2_CO_3_ 6% (5 ml) added, and were subsequently centrifuged (3000 × g, 5 min, 4°C). The upper toluene phase containing the fatty acid methyl esters was collected and stored in a gas chromatograph vial at −20°C for analysis.

Methylated fatty acid samples were analysed by gas chromatography using a fixed carbon-silica column 30 m × 0.25 mm (DB-225) (J&W Scientific, Folsom, CA, USA). The gas chromatograph was equipped with a flame ionization detector, autosampler, and autodetector. Injector and detector ports were set at 250°C. Oven temperature was programmed: 170°C for two minutes, increased 10°C/minute up to 190°C, where it remained constant for one minute. Temperature then increased 3°C/minute up to 220°C, which was maintained for a total run time of 30 minutes per sample. A split ratio of 10:1 and an injection volume of 3 μl were used. A known fatty acid mixture was compared to analysed samples to identify peaks according to retention time; their concentration was determined using a 6890 Series gas chromatograph (Hewlett Packard, Palo Alto, CA, USA) with Chemstations Version A. 04.02 (Chemstations Inc, Houston, TX, USA).

### Statistical analyses

The omega-3 index was calculated by adding erythrocyte EPA and DHA % (weight/weight) values^21^. Stratified analyses were carried out separating participants into tertiles according to omega-3 index. Thus, we compared participants in the two lower omega-3 index tertiles (LOI) with those in the highest tertile (HOI). Potential demographic differences between groups were assessed using one-way ANOVA and non-parametric Kruskal-Wallis test, in Minitab v.16 (Pennsylvania State University, State College, PA, USA). Random-effects mixed models with repeated measures were used to compare outcomes of interest between LOI and HOI men, using SAS v.9.3 (SAS Institute, Cary, NC, USA). All models accounted for important confounding factors, namely age, total body fat percentage, socioeconomic status (NZDep2006), physical activity levels (IPAQ), and the amount of saturated fat consumed. Birth order was also controlled for when assessing outcomes associated with glucose homeostasis and ambulatory blood pressure. Similar multivariate models and simple correlations were also used to test for linear associations with omega-3 index. All statistical tests were two-tailed and significance level maintained at 5%. Note that pairwise correlations between the independent variables were assessed, and none met the lowest threshold criteria for collinearity (r > 0.5) as described in Dormann et al.^34^. Parameters of glucose homeostasis and inflammatory markers were log-transformed to approximate normality. Demographic data are presented as means ± standard deviations; other data are means and 95% confidence intervals, adjusted for the confounders in multivariate models.

## Results

Forty seven men aged 46.5 ± 5.1 years and of BMI 27.4 ± 1.8 kg/m^2^ were studied. The majority of participants (87%) were of European descent.

### Linear associations

**Figure 1:**
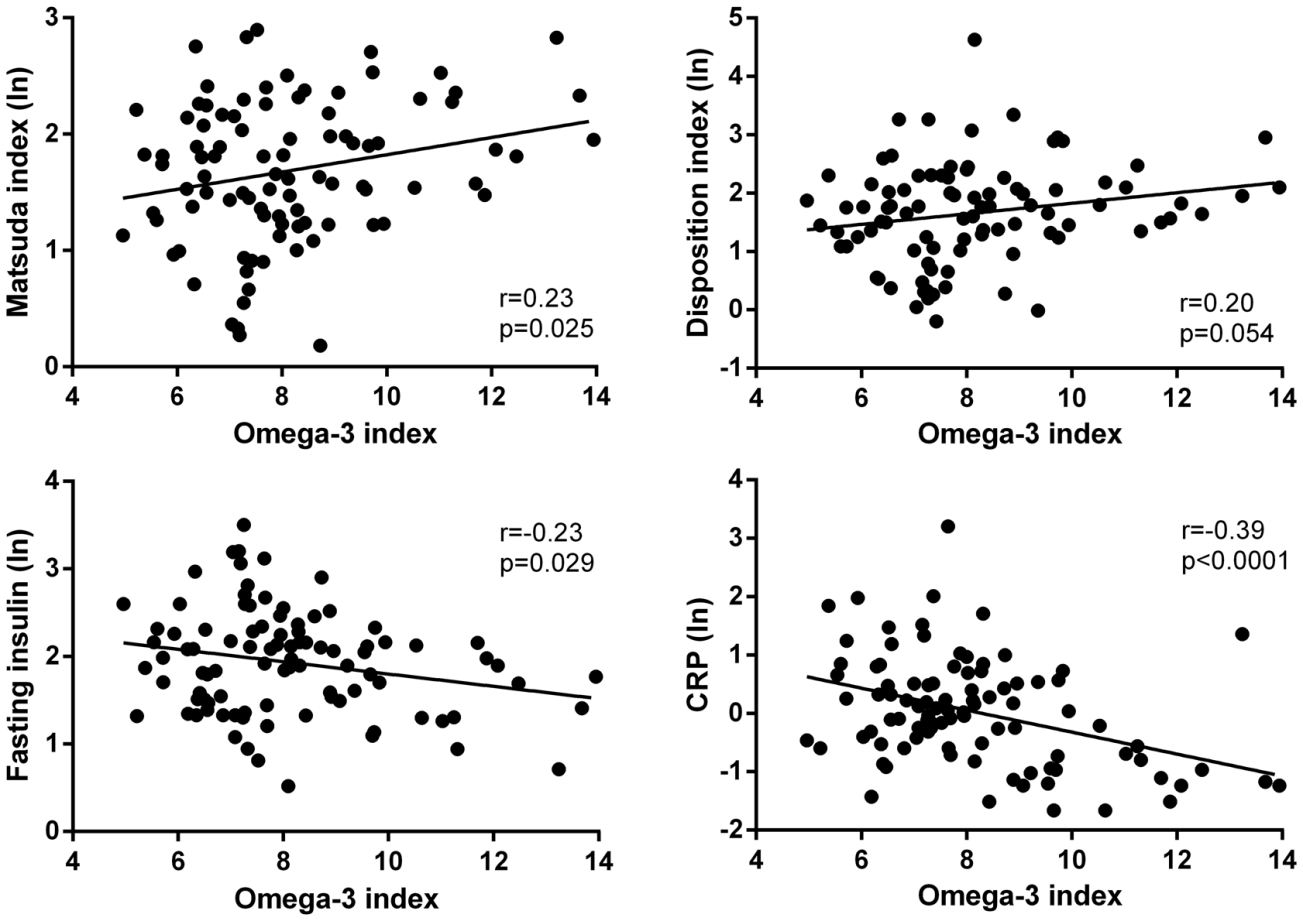
Linear associations between omega-3 index and parameters of glucose homeostasis and CRP.

Increasing omega-3 index was correlated with higher insulin sensitivity (r = 0.23; p = 0.025), higher disposition index (r = 0.20; p = 0.054), lower fasting glucose concentrations (r = −0.23; p = 0.029) (Fig. 1), and lower nocturnal systolic blood pressure (r = −0.21; p = 0.047). Multivariate analyses showed that increasing omega-3 index tended to be associated with higher insulin sensitivity (p = 0.079) and lower nocturnal systolic (p = 0.077) and diastolic (p = 0.073) blood pressure. Importantly, increasing omega-3 index was correlated with lower CRP concentrations (r = −0.39; p < 0.0001) (Fig. 1), and this association was corroborated by multivariate analysis (p = 0.049).

### Stratified analyses

Omega-3 index in HOI (n = 16) men were 42% higher than in LOI (n = 31) group (p < 0.0001; Table 1). HOI men had higher red cell phospholipid concentrations of oleic acid (p = 0.040), EPA (p < 0.0001), and DHA (p < 0.0001) than LOI men (Table 1). In contrast, HOI men had lower concentrations of stearic acid (p = 0.001), linoleic acid (p = 0.004), dihomo-γ-linolenic acid (p = 0.001), and arachidonic acid (p < 0.0001) (Table 1).

Participants in both groups were of similar age and socioeconomic status, had similar energy intake and consumption of saturated fat, and also engaged in comparable levels of physical activity (Table 2). However, HOI men were slightly leaner, by a difference of 1.1 kg/m^2^ in BMI (p = 0.041; Table 2). This difference highlights the importance of controlling for body fat (amongst other confounders) in all subsequent multivariate analyses.

Insulin sensitivity was 43% higher in HOI than in LOI men (Matsuda index 6.83 vs 4.78; p = 0.009) (Table 3), despite adjustment for important confounders that also affected insulin sensitivity, including age (p = 0.005), socioeconomic status (p < 0.0001), birth order (p = 0.017), and total body fat percentage (p = 0.010). The HOMA-IR index of insulin resistance corroborated these findings, showing that HOI men tended to be less insulin resistant than LOI men (p = 0.055; Table 3). In addition, fasting insulin concentrations were 25% lower in HOI than in LOI men (p = 0.038; Table 3). Consistent with improved insulin sensitivity, free fatty acids were 21% lower (p = 0.024) in HOI than in LOI men. In addition, the disposition index was 70% higher in HOI men (p = 0.013; Table 3), however there were no differences in fasting active GLP-1 (not shown) or the active GLP-1 response during the oral glucose tolerance test (Table 3).

HOI men displayed a more favourable blood pressure profile in the night time (Table 3). Thus, participants with the highest omega-3 index displayed lower nocturnal systolic blood pressure (−6.0 mmHg; p = 0.025), with a similar trend for nocturnal diastolic blood pressure (−3.5 mmHg; p = 0.072) (Table 3). HOI men also had better (greater) nocturnal systolic dip (p = 0.039; Table 3). There were however, no significant differences in daytime blood pressure (Table 3).

Men in the HOI group displayed 41% lower CRP concentration (p = 0.033) suggesting reduced systemic inflammation (Table 3). There were no differences in carotid-intima media thickness or lipid profile (Table 3).

### n-6 PUFA and n-6:n-3 ratio

Exploratory analyses were subsequently carried out to identify possible associations between n-6 PUFA concentrations and the n-6:n-3 ratio with metabolic parameters. Multivariate models yielded no significant associations between either n-6 or n-6:n3 ratio with any outcome response. Stratified analyses also compared the highest tertiles of n-6 PUFA and n-6:n-3 ratio with their respective lower tertiles. There were no observed differences for n-6, but the tertile with lowest n-6:n-3 ratio had lower CRP concentrations than the tertile with the highest ratio (0.85 (95%CI 0.63–1.13) vs 1.49 (95%CI 1.00–2.22) mg/l: p = 0.032).

## Discussion

This study showed that within a cohort of overweight middle-aged men, increasing omega-3 index was associated with greater insulin sensitivity and a more favourable metabolic profile. The tertile with highest omega-3 index had insulin sensitivity that was 43% greater than the two lower tertiles. In addition, they had improved β-cell function (greater oral disposition index), lower free fatty acid and CRP concentrations, lower night time systolic blood pressure and greater nocturnal systolic dipping.

A favourable metabolic profile in individuals with higher omega-3 index may be associated with lower risk of type 2 diabetes, the metabolic syndrome, and cardiovascular disease. Notably, the omega-3 index is a reliable measure of n-3 PUFA intake over several months^27^, but does not necessarily indicate sustained exposure in the longer-term. Thus, it is possible that some participants in the HOI group have not had sustained n-3 PUFA intake over the preceding years, which would be required for long-term metabolic and cardiovascular changes. Therefore, only differences in dynamic outcomes (e.g. insulin sensitivity, CRP and free fatty acid concentrations and blood pressure) would be expected, as observed in our subjects.

To the best of our knowledge, the association between increasing omega-3 index and improved insulin sensitivity has not been previously shown in adulthood. However, in line with our results, a study has reported a relationship between higher omega-3 index and lower HOMA-IR (a proxy of insulin resistance) in obese children^35^.

The reduction in insulin sensitivity seen with obesity appears to be due to adipose tissue expansion and inflammation, leading to abnormal adipose endocrine function and release of excess free fatty acids into circulation^36^,^37^,^38^. Raised free fatty acids have a central role in the development of both hepatic and muscle insulin resistance^36^, as well as important pro-inflammatory effects^39^. In our cohort, the effects of n-3 PUFA on insulin sensitivity may be mediated through reduction of free fatty acids. Long-chain n-3 PUFA are agonists of the nuclear transcription factors PPAR-α in liver^40^,^41^, as well as PPAR-γ^41^ and the recently characterised G-protein linked receptor GPR-120^11^ in adipose tissue. Thiazolidinediones are also PPAR-γ agonists, and may provide insight into the effect of n-3 PUFA via this nuclear receptor. Through PPAR-γ, thiazolidinediones increase the concentration of high molecular weight adiponectin^42^, reduce adipose tissue inflammation, and cause differentiation of preadipocytes^43^. In turn, this improves adipose tissue storage function and reduces the release of free fatty acids^43^.

In rats, n-3 PUFA appears to increase insulin sensitivity primarily through GPR-120, which inhibits adipose tissue inflammation via the NF-κB pathway^11^. However, the importance of this mechanism to the anti-inflammatory actions of n-3 PUFA is controversial^44^. Adipose tissue inflammation could also be reduced by changes in the balance of inflammatory and pro-resolving eicosanoids^45^. Importantly, these resolvins (enzymatic oxidation products of n-3 PUFA) are significantly more potent agonists of GPR120 and PPARγ than their parent fatty acids^44^, suggesting they may have an important role in mediating the increased insulin sensitivity associated with higher omega-3 index. Our data are consistent with these mechanisms, as higher concentrations of n-3 PUFA were associated with lower concentrations of free fatty acids and CRP (an inflammatory marker).

Our results are at odds with systematic reviews and large randomized controls examining the influence of supplemental n-3 PUFA on glycosylated haemoglobin (HbA1c) in adults with type 2 diabetes. The ORIGIN study examined the effects of n-3 PUFA and glargine (a basal insulin) in a large cohort made up mostly of adults with type 2 diabetes^46^. There were no effects of n-3 PUFA on cardiovascular outcomes or HbA1c. However, 50% of subjects were randomized to basal insulin and 59% were taking at least one oral glucose-lowering medication. Further, the majority were hypertensive and had a history of myocardial infarction or stroke. As such, study participants had significant metabolic dysfunction, morbidity, and cotreatment. Systematic reviews have shown that n-3 PUFA do not affect glycaemic control in diabetes^47^,^48^. However, adults with type 2 diabetes have more severe metabolic dysfunction than our participants who were simply overweight. We speculate that interrelated pathophysiological processes in type 2 diabetes (e.g. accumulation of intracellular lipid in liver and muscle and impaired insulin secretion) interact synergistically, such that n-3 PUFAs may reduce adipose inflammation and free fatty acids but do not influence the glycaemic control of diabetics.

The importance of the n-6:n-3 ratio to health outcomes is still debatable. Determining the relative importance of this ratio versus raw n-3 PUFA concentrations is difficult because they are not independent; supplementing n-3 PUFA leads to both an increase in n-3 levels and a decrease in n-6:n-3 ratio. It is logical to hypothesise that the agonist functions of n-3 PUFA would be proportional to n-3 concentration itself, but where n-3 PUFA is in direct competition with n-6 PUFA (such as for production of eicosanoids), the ratio of these PUFA subtypes could be more important. Our findings are in agreement with this hypothesis, as both lower n-6:n-3 ratio and increasing omega-3 index were associated with lower CRP concentrations, which was likely due to an altered balance of pro-inflammatory n-6-derived and pro-resolving n-3-derived eicosanoids. Importantly, the n-6:n-3 ratio was not associated with outcomes related to insulin sensitivity, which are more likely mediated through n-3 PUFA agonism of PPAR-γ and GPR-120.

The omega-3 index was also positively associated with the disposition index in both continuous and stratified analyses, suggesting an improvement in β-cell function with increasing omega-3 index. The oral disposition index has been shown to be predictive of future development of type 2 diabetes^49^. The mechanism for a relationship between omega-3 index and disposition index is unclear. Activation of GPR120 by free EPA and DHA in gut enteroendocrine cells leads to an increase in GLP-1, which enhances glucose-stimulated insulin secretion^50^. However, there was no association between the omega-3 index and GLP-1 in plasma during the oral glucose tolerance test. A direct effect of EPA and DHA on the β-cell is also possible and is consistent with our data. Although activation of GPR40 on the β-cell surface has a direct and positive effect on insulin secretion^51^, GPR40 is unlikely to mediate this effect as it is more potently activated by saturated rather than unsaturated fatty acids. It is plausible that GPR120 is also expressed on the β-cell surface, as GPR120 mRNA has been found within β-cells. However, evidence for the GPR120 receptor on the β-cell surface or a direct physiological role of GPR120 on insulin secretion is lacking^52^.

We also observed an association between higher omega-3 index and lower night time blood pressure and greater nocturnal systolic dipping. In particular, reduction in the normal systolic blood pressure dipping during sleep is an independent risk factor for cardiovascular disease^53^. As these effects were observed while the participants were asleep, they would not have been identifiable without 24-hour ambulatory measurement. It is not surprising that we did not detect a relationship between the omega-3 index and daytime blood pressure. The reported effects of n-3 PUFA on daytime blood pressure are subtle (reduction of systolic blood pressure by approximately 2 mmHg)^54^, and in our relatively small cohort they are likely to have been confounded by the effects of daily stress and activity.

Our ability to show an association between higher omega-3 index and improved insulin sensitivity is likely due to the relatively high plasma concentrations of n-3 PUFA observed in our participants. In our study, mean omega-3 index was 7.1% in the two lower tertiles, which would place the majority of these participants in the highest quartile of the omega-3 index reported in the Physicians' Health Study^55^. Thus, we speculate that n-3 PUFA concentrations in participants from previous studies^56^,^57^,^58^ were not sufficiently high to lead to detectable improvements in insulin sensitivity.

Within our cohort, the HOI group had lower BMI than the LOI group (a difference of 1.1 kg/m^2^). As body composition is an important determinant of insulin sensitivity^59^, it was paramount to consider body fat as a potential confounder, so that differences in adiposity were controlled for in our statistical analyses. An inverse association between adiposity and plasma n-3 PUFA^60^ or omega-3 index has been previously observed^61^, but there is no evidence that increased fat mass causes a reduction in circulating omega-3 levels. On the other hand, there is evidence from animal models that omega-3 supplementation leads to a reduction in adiposity, but the human evidence is limited and conflicting^62^. Nonetheless, a recent systematic review observed a small effect of omega-3 supplementation on body composition; participants taking fish oil lost 0.59 kg more than controls, with a reduction in BMI of 0.24 kg/m^2^^63^. As this effect was small, it is unlikely that the differences in BMI between groups in our study were a result of the differences in omega-3 index.

In this study, we have controlled for important confounders that are known to affect insulin sensitivity and other metabolic outcomes, including saturated fat consumption, physical activity levels, socioeconomic status, total body fat, and age. Further, the improved insulin sensitivity in the highest omega-3 index tertile was associated with lower free fatty acids, in fitting with the proposed mechanisms of action.

However, our study has important limitations. As this was an observational study, our findings are not sufficient to establish a causative relationship between omega-3 index and the metabolic differences identified. It is possible that other unknown factors (not controlled for in our statistical models) accounted for both the higher n-3 PUFA concentrations and the improved metabolic indices in the highest tertile. As we had a relatively low number of participants (n = 47) there was insufficient power to better examine a dose/response relationship. Lastly, we studied a relatively narrow range of individuals (overweight middle-aged males living in a large urban centre, mostly of New Zealand European ethnicity), which may limit wider applicability of our findings, particularly to women and to persons not in the overweight category.

In conclusion, higher n-3 PUFA concentrations in red cell phospholipids were associated with improved insulin sensitivity, lower free fatty acid and CRP concentrations, as well as improved nocturnal blood pressure profile in a group of overweight middle-aged men. Randomized controlled trials (that carefully control for potential confounders such as diet, physical activity, and body composition) are required to adequately investigate the effects of supplementation with long-chain n-3 PUFA on insulin sensitivity and metabolism.

## Figures and Tables

**Table 1:** Fatty acid profile in overweight middle-aged men in the upper tertile of omega-3 index (HOI) compared to those in the two lower tertiles (LOI). All data are % wt/wt in erythrocyte membrane fatty acids and are presented as means ± standard deviations. Omega-3 index represents the sum of EPA + DHA concentrations. Statistically significant values at p < 0.05 are shown in bold

	LOI	HOI	p-value
**n**	31	16	
**C16:0 (palmitic acid)**	22.5 ± 0.8	22.4 ± 0.6	0.60
**C16:1n-7 (palmitoleic acid)**	0.42 ± 0.14	0.41 ± 0.15	0.80
**C18:0 (stearic acid)**	18.7 ± 0.8	18.1 ± 0.7	**0.001**
**C18:1n-9 (oleic acid)**	14.3 ± 0.8	14.7 ±0.6	**0.040**
**C18:1n-7 (cis-vaccenic acid)**	1.38 ± 0.20	1.37 ± 0.12	0.60
**C18:2n-6 (linoleic acid)**	10.3 ± 1.4	9.5 ± 0.7	**0.004**
**C18:3n-6 (γ-linolenic acid)**	0.23 ± 0.10	0.21 ± 0.07	0.56
**C18:3n-3 (α-linolenic acid)**	0.17 ± 0.08	0.34 ± 0.86	0.37
**C20:0 (arachidic acid)**	0.43 ± 0.22	0.41 ± 0.19	0.64
**C20:1n-9 (eicosenoic acid)**	0.32 ± 0.18	0.34 ± 0.15	0.47
**C20:2n-6 (eicosadienoic acid)**	0.38 ± 0.21	0.42 ± 0.20	0.27
**C20:3n-6 (dihomo-γ-linolenic acid)**	2.25 ± 0.33	1.97 ± 0.43	**0.001**
**C20:4n-6 (arachidonic acid)**	17.8 ± 1.2	15.7 ± 1.1	**<0.0001**
**C20:5n-3 (eicosapentaenoic acid)**	1.27 ± 0.36	2.10 ± 0.66	**<0.0001**
**C22:5n-3 (docosapentaenoic acid)**	3.78 ± 0.53	4.00 ± 0.59	0.073
**C22:6n-3 (docosahexaenoic acid)**	5.83 ± 0.80	8.02 ± 1.26	**<0.0001**
**Omega-3 index**	7.1 ± 0.8	10.1 ± 1.5	**<0.0001**

**Table 2:** Characteristics of the study population, consisting of overweight middle-aged men in the upper tertile of omega-3 index (HOI) compared to those in the two lower tertiles (LOI). Data are means ± standard deviations

	LOI	HOI	p-value
n	31	16	
Age (years)	46.0 ± 5.0	47.4 ± 5.3	0.38
BMI (kg/m^2^)	27.8 ± 1.7	26.7 ± 1.7	0.041
Socioeconomic status (NZDep2006)	4.0 ± 2.5	3.9 ± 2.0	0.88
Physical activity levels (IPAQ)	4322 ± 4058	4442 ± 4038	0.75
Total energy intake (kJ/day)	9150 ± 3043	10063 ± 2202	0.29
Saturated fat intake (g/day)	30.4 ± 13.7	31.1 ± 8.6	0.86

**Table 3:** Study outcomes in overweight middle-aged men in the upper tertile of omega-3 index (HOI) compared to those in the two lower tertiles (LOI). Data are means and 95% confidence intervals adjusted for confounding factors in the multivariate models, including the repeated measures within each participant. GLP-1 AUC represents the area under the curve measured during a 120-minute oral glucose tolerance test. Statistically significant values at p < 0.05 are shown in bold

	LOI	HOI	p-value
**n**	31	16	
**Glucose homeostasis**			
Insulin sensitivity (Matsuda index)	4.78 (4.14–5.52)	6.83 (5.53–8.43)	**0.009**
Disposition index	4.37 (3.50–5.47)	7.44 (5.34–10.37)	**0.013**
HOMA-IR	1.82 (1.58–2.11)	1.41 (1.14–1.74)	0.055
Fasting glucose (mmol/l)	5.39 (5.19–5.59)	5.35 (5.21–5.49)	0.73
Fasting insulin (mU/l)	7.46 (6.43–8.66)	5.60 (4.50–6.97)	**0.038**
**24-hour ambulatory blood pressure**			
Daytime systolic (mmHg)	126.6 (123.2–130.1)	124.8 (119.7–130.0)	0.57
Daytime diastolic (mmHg)	80.0 (77.4–82.6)	78.1 (74.3–82.0)	0.43
Night time systolic (mmHg)	112.6 (109.8–115.5)	106.6 (102.4–110.9)	**0.025**
Night time diastolic (mmHg)	67.7 (65.6–69.7)	64.2 (61.2–67.3)	0.072
Systolic dip (%)	10.8 (8.8–12.8)	14.7 (11.7–17.6)	**0.039**
Diastolic dip (%)	15.4 (12.9–17.8)	17.7 (14.1–21.4)	0.30
**Carotid intima-media thickness** (mm)	0.80 (0.75–0.84)	0.84 (0.77–0.91)	0.30
**Other metabolic markers**			
Free fatty acids (mmol/l)	0.38 (0.34–0.43)	0.30 (0.26–0.36)	**0.024**
Uric acid (umol/l)	360 (337–384)	354 (321–387)	0.77
CRP (mg/l)	1.16 (0.88–1.55)	0.69 (0.48–1.01)	**0.033**
Active GLP-1 (AUC)	1129 (913–1346)	950 (645–1255)	0.35
**Lipid profile**			
Total cholesterol (mmol/l)	5.16 (4.92–5.40)	4.94 (4.59–5.29)	0.31
LDL-C (mmol/l)	3.51 (3.28–3.73)	3.41 (3.09–3.73)	0.64
HDL-C (mmol/l)	1.05 (0.97–1.13)	1.05 (0.94–1.18)	0.92
Total cholesterol: HDL-C	4.86 (4.46–5.30)	4.63 (4.10–5.23)	0.52
Triglycerides (mmol/l)	1.14 (1.00–1.29)	1.01 (0.85–1.22)	0.31
